# Profilin 1 with the amyotrophic lateral sclerosis associated mutation T109M displays unaltered actin binding and does not affect the actin cytoskeleton

**DOI:** 10.1186/s12868-015-0214-y

**Published:** 2015-11-16

**Authors:** Axel Freischmidt, Marcel Schöpflin, Marisa S. Feiler, Ann-Katrin Fleck, Albert C. Ludolph, Jochen H. Weishaupt

**Affiliations:** Department of Neurology, Ulm University, Albert-Einstein-Allee 11, 89081 Ulm, Germany

**Keywords:** Amyotrophic lateral sclerosis, ALS, Profilin 1, PFN1, Neurodegeneration, Cytoskeleton, Actin

## Abstract

**Background:**

The recent identification of several mutations in PFN1, a protein involved in actin dynamics, strengthens the hypothesis that pathology of amyotrophic lateral sclerosis is linked to cytoskeletal defects. Impaired actin binding is a common denominator of several *PFN1* mutations associated with amyotrophic lateral sclerosis, although further mechanisms may also contribute to the death of motor neurons. In this study we examine the actin binding properties of PFN1 carrying the causal T109M mutation and its effects on the actin cytoskeleton.

**Methods:**

Actin binding of PFN1 T109M was examined by co-immunoprecipitation experiments, a split luciferase complementation assay and a pulldown assay with recombinant PFN1. The actin cytoskeleton was investigated by fluorescence microscopy and by ultracentrifuge separation of globular and filamentous actin fractions followed by Western blotting.

**Results:**

Using different technical approaches we show that PFN1 T109M displays unaltered actin binding. Furthermore we show that the actin cytoskeleton is not affected by PFN1 carrying the T109M mutation.

**Conclusion:**

Our data suggest that actin independent mechanisms contribute to the pathogenicity of *PFN1* T109M and possibly other *PFN1* mutations.

## Background

Familial cases of the fatal neurodegenerative disease amyotrophic lateral sclerosis (ALS) comprise about 10 % of all cases. Approximately two-thirds thereof can be explained by mutations in known ALS genes, with *C9orf72*, *SOD1*, *TARDBP* and *FUS* being the most frequent [1]. Recently, mutations in several other genes, e.g. *PFN1* [2] or *HNRNPA1* [3], have been shown to be a rare cause of ALS. Analysis of these rare causal mutations is a valuable tool for the uncovering of altered pathways possibly common to ALS pathogenesis.

The discovery that *PFN1* mutations cause ALS [2] implicates disturbed dynamics of the cytoskeleton in degeneration of motor neurons and are in accordance with previous reports associating mutations in *NEFH* [4], *PRPH* [5] or *DCTN1* [6] with ALS. PFN1 regulates dynamics of actin polymerization but also binds to a great variety of proteins serving as a platform involved in controlling multiple other cellular processes like membrane trafficking or even splicing events in the nucleus [7]. Recently, PFN1 was implicated in the formation and clearance of stress granules and seems to harbor an intrinsic ability to induce stress granule formation upon overexpression [8]. Stress granules are transient cellular structures impeding translation upon different stressors and are composed of RNA-binding proteins, mRNAs as well as factors involved in translation repression. Chronic cellular stress and a pathogenic transition of transient stress granules to permanent aggregates of RNA-binding proteins are thought to be important in ALS [9].

So far, eight missense mutations in *PFN1* have been associated with ALS, namely C71G, M114T, E117G, G118V [2], T109M [10], R136W [11], A20T and Q139L [12]. It has been shown that PFN1 proteins carrying the C71G, M114T or G118V substitutions are impaired in actin binding while the PFN1 E117G variant retains its ability binding to actin [2]. This finding parallels genetic evidences supporting monogenic pathogenicity of *PFN1* C71G, M114T and G118V while assigning the *PFN1* E117G variant the role of a risk factor for ALS [2, 12, 13]. So far, no biochemical data exists about the actin binding properties of PFN1 T109M, R136W, A20T and Q139L. While the *PFN1* R136W, Q139L and A20T mutations were detected in single individuals with sporadic or familial ALS, respectively [11, 12], pathogenicity of the *PFN1* T109M mutation is supported by co-segregation with the disease in the affected family [10] as well as serum microRNA profiles of pre-clinical mutation carriers that are highly similar to profiles of pre-clinical carriers of ALS associated mutations in *C9orf72* or *SOD1* [14].

In order to further elucidate the relevance of altered actin binding for ALS-associated PFN1 mutant proteins, we studied in detail the actin binding properties of the PFN1 T109M variant as well as its impact on the actin cytoskeleton.

## Results

### Unaltered expression and splicing of PFN1 T109M

**Fig. 1:**
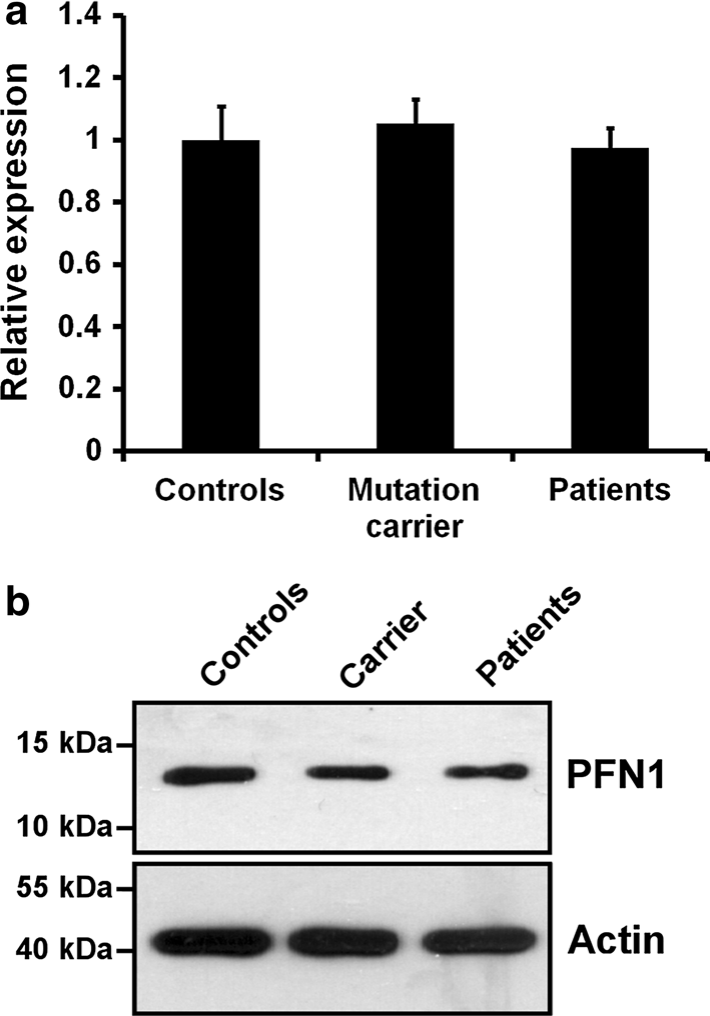
PFN1 protein or mRNA levels are not affected by the *PFN1* T109M mutation. **a** Measurement of relative PFN1 mRNA abundance by qRT-PCR of LCLs of five healthy controls and *PFN1* T109M mutant LCLs of two ALS patients and three pre-clinical mutation carriers. Results were normalized to U6 snRNA using 2^−ΔΔCt^-method (*bars* indicate mean ± SEM). **b** Western blot comparing amounts of PFN1 protein of LCLs of five healthy controls, two *PFN1* T109M mutant ALS patients and three pre-clinical carriers of the *PFN1* T109M mutation. Same amounts of protein were pooled in the respective group. Actin is shown as a loading control

The *PFN1* c.326C>T mutation causing the T109M substitution is located directly at an exon/intron boundary (first base of Exon 3) [10]. Although not predicted by splice site prediction software (e.g. http://www.fruitfly.org/seq_tools/splice.html), we wanted to rule out the possibility that pathogenicity of the *PFN1* T109M mutation is due to a haploinsufficiency caused by impaired splicing of the mRNA. We therefore measured relative *PFN1* mRNA abundance and PFN1 protein levels in LCLs of five healthy controls as well as in *PFN1* T109M mutant LCLs of two clinically definite ALS patients and three pre-clinical mutation carriers (Fig. 1). No significant difference of *PFN1* mRNA or PFN1 protein levels could be detected between the different groups.

### PFN1 T109M is not impaired in ß-actin binding

**Fig. 2:**
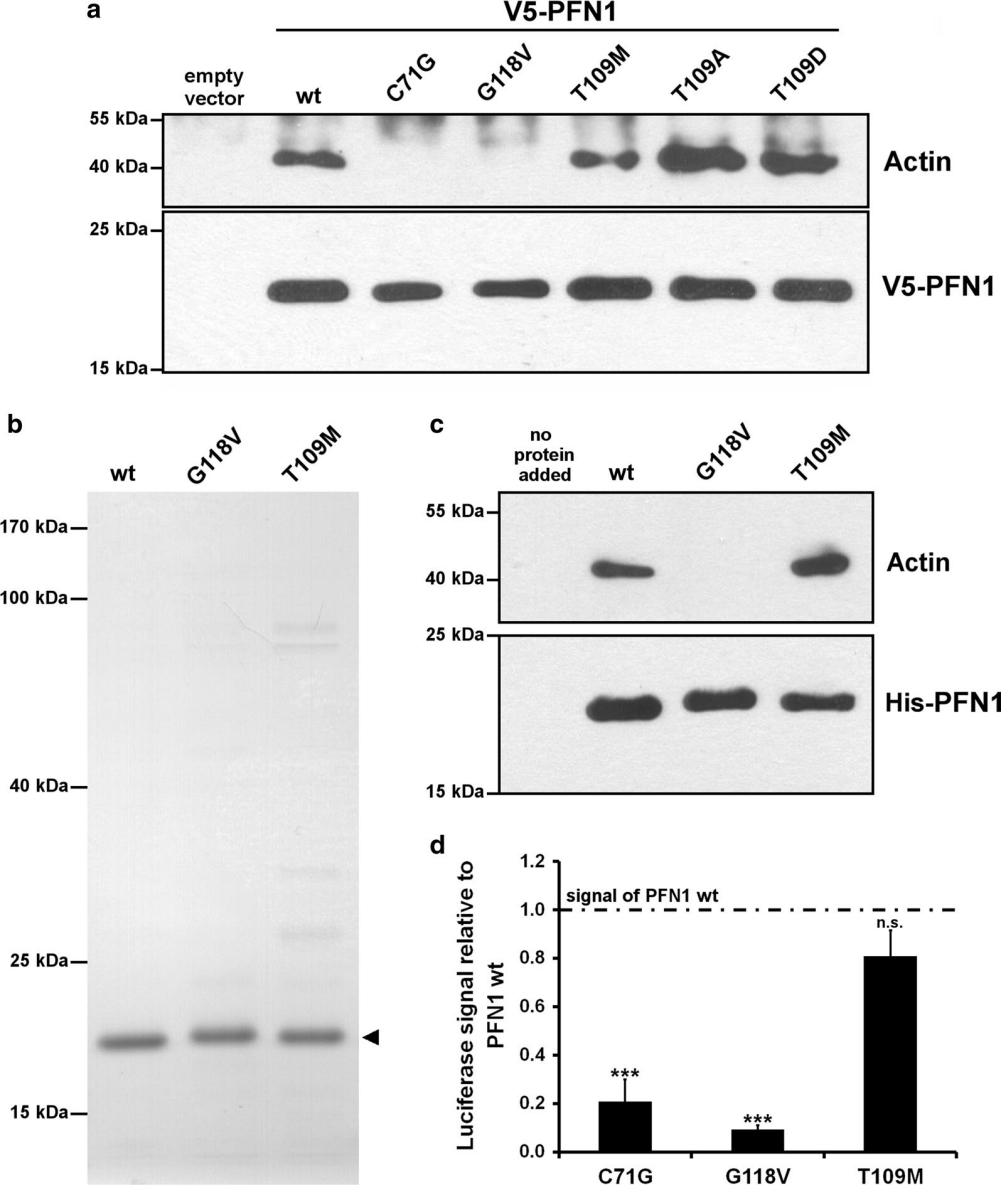
PFN1 T109M mutant protein is not impaired in actin binding. **a** Co-immunoprecipitation of lysates of HEK293 cells overexpressing wildtype and mutant V5-PFN1 using a V5-antibody followed by Western blot detection of V5-PFN1 and actin. **b** Purified recombinant His_6_-tagged wildtype and mutant PFN1 (*arrowhead*) used for the pulldown assay was subjected to SDS-PAGE followed by staining with coomassie brilliant blue. **c** His_6_-tag pulldown using recombinant His_6_-PFN1 and lysates of HEK293 cells. His_6_-tagged PFN1 and binding proteins were precipitated with Ni–NTA agarose (Qiagen) and subjected to Western blotting. **d** Quantification of the interaction of actin with wildtype and mutant PFN1 using a split luciferase assay. Luciferase activity from protein complementation was measured in HEK293 cells 24 h after transfection with plasmids coding for PFN1-hGluc(1) and actin-hGluc(2). Signals were normalized to the expression of both fusion proteins determined by Western blotting and are shown relative to the signal of wildtype PFN1-hGluc(1) and actin-hGluc(2) (n = 4; 2–7 measurements each; *bars* indicate mean ± SEM; *** *p* ≤ 0.001 in a two tailed *student’s t* test; n.s. = not significant)

The PFN1 T109M substitution is located in close spatial proximity to C71G, M114T, E117G and G118V near the actin binding surface of PFN1 [10]. Since PFN1 substitutions C71G, M114T and G118V but not E117G impair actin binding [2] we examined the actin binding properties of the PFN1 T109M mutant protein. Additionally, the T109 of PFN1 is a phosphorylation site occupied in vivo [15] and altered actin binding by disrupted phosphorylation of T109 was suggested before to possibly contribute to toxicity of this mutation [10]. We therefore performed a co-immunoprecipitation using a V5-antibody in lysates of HEK293 cells overexpressing V5-PFN1 wildtype or different V5-PFN1 mutant proteins including a phospho-deficient (T109A) and a phospho-mimicking (T109D) mutant. Western blot detection of co-precipitated actin revealed no deficits in the binding properties of PFN1 T109M (Fig. 2a). In contrast to the expected opposing effects of the PFN1 T109A and T109D mutant proteins on actin binding we found for both an increased affinity to actin. Hence, we omitted these artificial phospho-mimicking and phospho-deficient proteins in further experiments.

To validate the unchanged actin binding properties of PFN1 T109M we performed a pulldown assay using recombinant His_6_-PFN1 (Fig. 2b, c) and quantified the PFN1-actin interaction in living cells using a split luciferase complementation assay (Fig. 2d). Both approaches confirmed the unchanged actin binding properties of PFN1 T109M, while the previously reported reduction in actin binding of PFN1 C71G and G118V [2] were reproduced.

### The actin cytoskeleton of HEK293 cells is not affected by PFN1 T109M

**Fig. 3:**
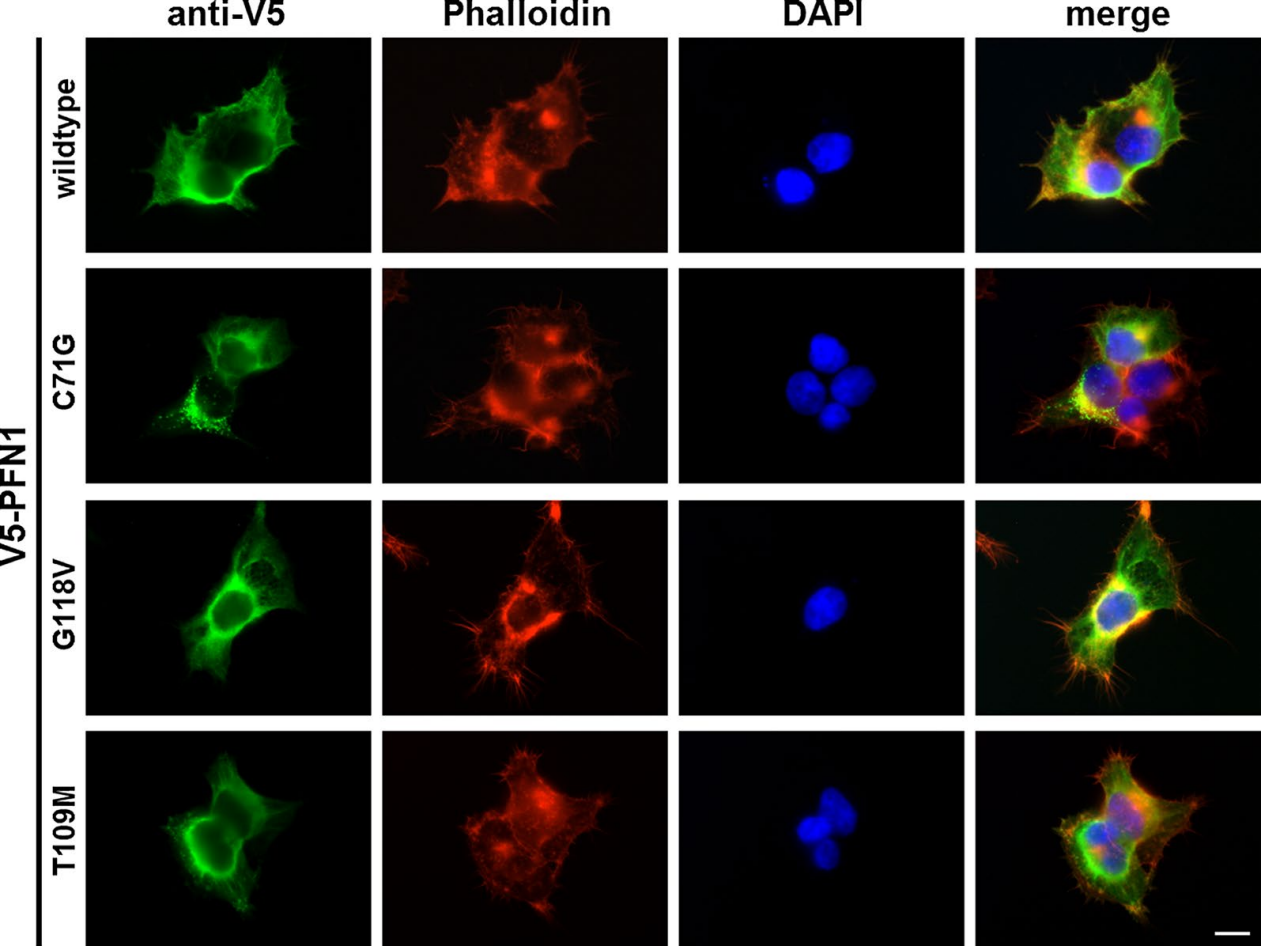
Unaltered actin cytoskeleton in HEK293 cells overexpressing wildtype and mutant V5-PFN1. HEK293 cells transfected with plasmids coding for wildtype and mutant V5-PFN1 were stained for exogeneous PFN1 (anti-V5) and endogeneous F-actin (Phalloidin). Note the formation of aggregates of the V5-PFN1 C71G protein in roughly 50 % of cells reported previously [2]. *Scale bar* is 10 µm

Besides binding of actin monomers, another important function of PFN1 affecting the polymerization of actin is the exchange of actin bound ADP to ATP (see [7] for a review). Hence, the unaltered actin binding properties of the PFN1 T109M mutant protein described above do not exclude an impact of this mutation on nucleotide exchange and actin polymerization. To address this issue, we stained filamentous actin (F-actin) in HEK293 cells overexpressing wildtype and mutant V5-PFN1 using Phalloidin (Fig. 3). We could not identify any marked differences in cell morphology and quantity or distribution of F-actin between wildtype and mutant V5-PFN1 expressing cells. Interestingly, even the PFN1 mutants C71G (in cells in which this aggregation-prone protein was not aggregated) and G118V, which are clearly impaired in actin binding, still co-localize with the dense F-actin structures near the plasma membrane (lamellipodia, filopodia and cortical F-actin).

**Fig. 4:**
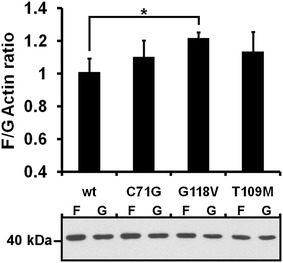
The T109M mutation of PFN1 does not change the F/G-actin ratio in HEK293 cells. *Upper panel* F/G-actin ratios of HEK293 cells overexpressing wildtype and mutant V5-PFN1. F- and G-actin fractions of the respective cell lysates were separated by ultracentrifugation and actin levels analyzed by Western blotting and densitometry (n = 5; *bars* indicate mean ± SEM; * *p* ≤ 0.05 in a two tailed *student’s t* test). *Lower panel* Representative Western blot of F- and G-actin fractions used for densitometric analysis

To further verify the observations of immunocytochemistry, we determined the ratio of filamentous and globular actin (F/G-actin ratios) in HEK293 cells overexpressing wildtype and mutant V5-PFN1 (Fig. 4). Also in this experiment no difference was detected between cells expressing V5-PFN1 wildtype and C71G (*p* = 0.502) or T109M mutant protein (*p* = 0.414). Solely V5-PFN1 G118V slightly (1.21-fold; *p* = 0.0471) increased the F/G-actin ratio compared to wildtype PFN1.

## Discussion

In this study, we show that ALS-associated PFN1 T109M mutant protein is not impaired in actin binding and does not affect the actin cytoskeleton using several different approaches. Furthermore, we exclude that pathogenicity of this mutation located directly at an exon/intron boundary is due to a splicing defect. Taken together, our results provide evidence for additional or alternative mechanisms beyond impairment of the actin cytoskeleton responsible for motor neuron degeneration associated with *PFN1*-linked ALS.

The finding that ALS-associated mutations in PFN1 are deficient in actin binding suggests disturbed actin dynamics to cause motor neuron degeneration [2]. However, our results show unchanged actin binding of the causal PFN1 T109M variant. Therefore, exclusion of haploinsufficiency by a splicing defect as the pathogenic mechanism of the *PFN1* T109M mutation, which is located close to an exon/intron boundary [10], is an important approach. We were not able to examine CNS tissue affected in ALS and thus cannot exclude splicing defects in neurons. However, unchanged PFN1 mRNA and protein levels in LCLs of ALS patients and pre-clinical carriers of the *PFN1* T109M mutation argue against haploinsufficiency as the pathogenic mechanism.

Phosphorylation of T109 of PFN1 was proposed to fine-tune actin binding and loss of this phosphorylation site by the T109M substitution might result in disturbed actin dynamics [10]. Increased actin binding of the phospho-mimicking PFN1 T109D mutant protein reported here would be in line with this hypothesis. However, since actin binding of the phospho-deficient PFN1 T109A was increased as well and did not show the opposite effect of the phospho-mimicking T109D mutation, we conclude that both artificial mutants are not suitable to examine the influence of phosphorylation on actin binding. Possibly both mutations induce structural changes in PFN1 increasing its affinity to actin. Nevertheless, the finding that the phosphorylation-deficient PFN1 T109M mutant protein exhibits no differences in actin binding compared to wildtype PFN1 (which is phosphorylated at T109 [15]) strongly argues for an independence of actin binding from the phosphorylation of T109 in PFN1.

Further evidence recently provided in yeast supports activity of PFN1 T109M mutant protein in actin dynamics. PFN1 T109M and E117G but not C71G, M114T and G118V were able to rescue the growth of yeast strains depleted in *Pfy1*, the yeast homolog of *PFN1* [8]. Hence, the ability to substitute for Pfy1 seems to parallel actin binding capability of PFN1. In the same study, a genetic screen in yeast identified stress granule formation to be closely connected to PFN1 and the recruitment to and clearance from stress granules of PFN1 and ALS-associated mutant PFN1 proteins as well as their impact on stress granule formation was studied in detail using various assays. Interestingly, regarding the intrinsic ability of PFN1 to induce stress granules upon overexpression or the clearance of stress granules from cells after heat shock, the PFN1 T109M mutant protein paralleled the characteristics of PFN1 C71G, M114T and G118V more closely than of PFN1 E117G [8]. Hence, recruitment to and clearance from stress granules of PFN1 variants seems to be more closely connected to pathogenicity than the ability to bind actin. The only exception is the recruitment of mutant PFN1 to arsenite-induced stress granules. Here, a similar recruitment of PFN1 T109M and E117G as well as a decreased recruitment of C71G, M114T and G118V indicate an actin dependent mechanism. Further studies are thus warranted to evaluate a possible involvement of stress granules in *PFN1*-linked neurodegeneration in more details.

Additional to unchanged actin binding, we show that PFN1 T109M has no apparent effect on the actin cytoskeleton and does not alter the F/G-actin ratio in HEK293 cells. We therefore assume that PFN1 T109M is fully functional concerning its interactions with actin, i.e. binding and catalysis of nucleotide exchange. However, our study does not exclude defects of actin dynamics caused by PFN1 T109M, e.g. the rate of reorganization of the actin cytoskeleton in response to stimuli. Interestingly, overexpression of PFN1 C71G and G118V also had no apparent effect on the actin cytoskeleton and only PFN1 G118V significantly increased the F/G-actin ratio in HEK293 cells. These observations are in line with recent results of Boopathy and colleagues [16] showing that recombinant PFN1 C71G, M114T and E117G similarly to wildtype PFN1 reduce the elongation rate of actin filaments in an in vitro polymerization assay. Solely PFN1 G118V showed a trend towards impairment of this function. Hence, only PFN1 G118V seems to have an impact on actin polymerization that is different from the wildtype protein.

Taken together, increasing evidence indicates the involvement of other/additional mechanisms in *PFN1*-linked ALS than impairments of the actin cytoskeleton. For example, mutation-induced destabilization of PFN1 has been suggested [16]. Taking into account the multi-functionality of PFN1 [7], several additional mechanisms are possible. For example, PFN1 interacts directly with SMN, a key-player in spinal muscular atrophy (see e.g. [17] for a review), and both co-localize in nuclear gems [18]. Nuclear gems are sub-nuclear structures involved in mRNA splicing events and depletion of nuclear gems has been repeatedly reported in ALS (reviewed in [19]). Mutant PFN1 could thus influence the regulation of such protein/RNA granules. Further studies and analysis of additional ALS-associated PFN1 mutant proteins are urgently needed to better understand the mechanisms ultimately resulting in the death of motor neurons.

## Conclusions

This study provides strong evidence that binding of the ALS-associated PFN1 T109M mutant protein to actin is not impaired and that this mutation has no effect on the actin cytoskeleton, suggesting additional or alternative mechanisms for *PFN1*-linked neurodegeneration beyond an impairment of cytoskeletal dynamics.

## Methods

### Patient cohorts and ethics statement

Appropriate approval and procedures were used concerning human subjects. Blood samples were drawn with prior informed written consent according to the Declaration of Helsinki (WMA, 1964) approved by the national medical ethical review boards. Anonymous genotyping by sequencing of the *PFN1* gene was performed as previously described [10].

### Analysis of lymphoblastoid cell lines (LCLs)

Epstein-Barr virus transformed LCLs were generated from healthy controls, *PFN1* T109M mutant ALS patients as well as pre-clinical mutation carriers.

cDNA of LCLs was used for quantitative real-time PCR (qRT-PCR) measurements of *PFN1* mRNA run on a CFX96 Real-Time System (Bio-Rad) using oligonucleotides spanning Exons 2 and 3: 5′-GCCAGAAATGTTCGGTGATC-3′ and 5′-CTTTGCCCATCAGCAGGAC-3′. Results were normalized relative to U6 snRNA [20] using 2^−ΔΔCt^-method [21].

Lysates of LCLs were subjected to Western blotting using rabbit anti-ß-actin (Cell Signaling Technology Cat# 4970 RRID:AB_2223172) and rabbit anti-PFN1 (Sigma-Aldrich Cat# P7749 RRID:AB_1079599) antibodies. Secondary antibody was goat anti-rabbit IgG-HRP (Life Technologies Cat# G21234 RRID:AB_10837906).

### Plasmids

Eukaryotic expression vector of human N-terminally V5-tagged PFN1 [GenBank:NM_005022.3] was pcDNA3.1/nV5-DEST (Invitrogen) previously described [2]. PFN1 T109M, T109A and T109D mutations were introduced using site directed mutagenesis (Table 1).

**Table 1:** Oligonucleotides used for generation of PFN1 T109M, T109A and T109D by site directed mutagenesis

Mutation	Sequence
T109M	5′-CTGTCACCAAGACTGACAAGATGCTAGTCCTGCTGATGGGC-3′
	5′-GCCCATCAGCAGGACTAGCATCTTGTCAGTCTTGGTGACAG-3′
T109A	5′-CTGTCACCAAGACTGACAAGGCGCTAGTCCTGCTGATGGGC-3′
	5′-GCCCATCAGCAGGACTAGCGCCTTGTCAGTCTTGGTGACAG-3′
T109D	5′-CTGTCACCAAGACTGACAAGGATCTAGTCCTGCTGATGGGC-3′
	5′-GCCCATCAGCAGGACTAGATCCTTGTCAGTCTTGGTGACAG-3′

Expression vectors for the split luciferase complementation assay were pcDNA3.1-Zipper-hGluc(1) and pcDNA3.1-Zipper-hGluc(2) [22] previously used for detection of α-synuclein oligomers [23]. The coding sequence of α-synuclein was replaced by the coding sequence of human ß-actin [GenBank:NM_001101.3] or PFN1 (wt or mutants), resulting in expression constructs coding for PFN1-hGluc(1) and actin-hGluc(2).

For expression of N-terminally His_6_-tagged recombinant PFN1 in *E. coli* we used pRSET B vector (life technologies).

### Cultivation and transfection of HEK293 cells

HEK293 cells were cultivated in DMEM + 10 % FCS (Gibco). Transfections were carried out using the calcium-phosphate method as described before [24] with minor modifications.

### Co-immunoprecipitation

Co-immunoprecipitation of ß-actin with V5-PFN1 in lysates of HEK293 cells was performed as previously described [2]. For Western blot detection of V5-PFN1 and ß-actin we used mouse anti-V5 (Life Technologies Cat# R96025 RRID:AB_10050879) and rabbit anti-ß-actin (Cell Signaling Technology Cat# 4970 RRID:AB_2223172) antibodies. Secondary antibodies were goat anti-mouse IgG-HRP (Life Technologies Cat# G21040 RRID:AB_11180340) and goat anti-rabbit IgG-HRP (Life Technologies Cat# G21234 RRID:AB_10837906), respectively.

### Split luciferase complementation assay

Luciferase activity from protein complementation was measured in HEK293 cells co-transfected with PFN1-hGluc(1) and actin-hGluc(2) 24 h post-transfection as previously described [23] using Multilabel Reader Victor X3 (Perkin Elmer). Luciferase signal was normalized to expression of PFN1-hGluc(1) and actin-hGluc(2) determined by Western blotting.

### Expression and purification of His_6_-PFN1 in *E. coli*

His_6_-tagged PFN1 was expressed for 4 h upon IPTG-induction (1 mM) at 37° C in *E. coli* BL21(DE3)pLysS (Novagen). Purification of recombinant PFN1 was performed under native conditions in a batch mode using Ni–NTA agarose (Qiagen) as specified by the manufacturer.

### His_6_-PFN1 pulldown

HEK293 lysates in RIPA buffer were incubated for 1 h with His_6_-PFN1 at 4 °C with constant rotation. Ni–NTA agarose (Qiagen) equilibrated in RIPA buffer was added and further incubated for 1 h. Subsequently, the beads were washed four times with RIPA buffer + 10 mM imidazole and finally boiled in protein sample buffer + 200 mM imidazole. Western blot detection of His_6_-PFN1 and ß-actin was performed with antibodies already used for LCL analysis.

### Immunocytochemistry

24 h post-transfection, HEK293 cells overexpressing wildtype and mutant V5-PFN1 were washed in PBS, fixed for 10 min in Roti-Histofix 4 % (Roth), permeabilized for 10 min in PBS supplemented with 0.1 % Triton X-100 and 100 mM glycine and blocked for 45 min in PBS with 1.5 % bovine serum albumin and 0.1 % Tween20. Afterwards, cells were incubated for 1 h with the primary antibody mouse anti-V5 (see above; 1:750 in PBS), washed in PBS and incubated for 1 h with the secondary antibody goat anti-mouse DyLight 488 (Thermo Fisher Scientific Cat# 35502 RRID:AB_844397; 1:500 in PBS) and Phalloidin-Atto 594 (Sigma-Aldrich Cat# 51927; 600 nM in PBS) followed by washing in PBS. Cell nuclei were stained for 10 min using DAPI (1 µg/ml in PBS). After a final washing in PBS, cells were mounted on microscope slides and analyzed using a Carl Zeiss Axio Observer.A1 microscope.

### Filamentous/globular (F/G) actin assay in HEK293 cells

24 h post-transfection, HEK293 cells overexpressing wildtype and mutant V5-PFN1 were washed in PBS and lysed at room temperature in F-actin stabilization buffer [50 mM PIPES, pH 6.9, 50 mM NaCl, 5 mM MgCl_2_, 5 mM EGTA, 5 % glycerol, 0.1 % NP-40, 0.1 % Triton X-100, 0.1 % Tween20, 0.1 % β-mercaptoethanol, 1 mM ATP, complete protease inhibitor (Roche)]. After adjustment to a total protein concentration of 2 mg/ml, lysates were centrifuged at 100,000×*g* and 25° C for 1 h and supernatants were transferred to a new tube representing the G-actin fraction. The pellets (F-actin fraction) were completely dissolved in the initial volume of solubilization buffer [10 mM Tris, pH 8.0, 150 mM NaCl, 2 % SDS, complete protease inhibitor (Roche)] by three subsequent cycles of sonication, heating to 70 °C for 10 min and cooling down to room temperature. Equal volumes of the F- and G-actin fractions were subjected to Western blotting followed by detection of ß-actin using antibodies described above. Western blots were densitometrically analyzed using ImageJ software.

## References

[1] Renton AE, Chio A, Traynor BJ (2014). State of play in amyotrophic lateral sclerosis genetics. Nat Neurosci.

[2] Wu CH, Fallini C, Ticozzi N, Keagle PJ, Sapp PC, Piotrowska K, Lowe P, Koppers M, McKenna-Yasek D, Baron DM (2012). Mutations in the profilin 1 gene cause familial amyotrophic lateral sclerosis. Nature.

[3] Kim HJ, Kim NC, Wang YD, Scarborough EA, Moore J, Diaz Z, MacLea KS, Freibaum B, Li S, Molliex A (2013). Mutations in prion-like domains in hnRNPA2B1 and hnRNPA1 cause multisystem proteinopathy and ALS. Nature.

[4] Al-Chalabi A, Andersen PM, Nilsson P, Chioza B, Andersson JL, Russ C, Shaw CE, Powell JF, Leigh PN (1999). Deletions of the heavy neurofilament subunit tail in amyotrophic lateral sclerosis. Hum Mol Genet.

[5] Gros-Louis F, Lariviere R, Gowing G, Laurent S, Camu W, Bouchard JP, Meininger V, Rouleau GA, Julien JP (2004). A frameshift deletion in peripherin gene associated with amyotrophic lateral sclerosis. J Biol Chem.

[6] Puls I, Jonnakuty C, LaMonte BH, Holzbaur EL, Tokito M, Mann E, Floeter MK, Bidus K, Drayna D, Oh SJ (2003). Mutant dynactin in motor neuron disease. Nat Genet.

[7] Witke W (2004). The role of profilin complexes in cell motility and other cellular processes. Trends Cell Biol.

[8] Figley MD, Bieri G, Kolaitis RM, Taylor JP, Gitler AD (2014). Profilin 1 associates with stress granules and ALS-linked mutations alter stress granule dynamics. J Neurosci.

[9] Li YR, King OD, Shorter J, Gitler AD (2013). Stress granules as crucibles of ALS pathogenesis. J Cell Biol.

[10] Ingre C, Landers JE, Rizik N, Volk AE, Akimoto C, Birve A, Hubers A, Keagle PJ, Piotrowska K, Press R (2013). A novel phosphorylation site mutation in profilin 1 revealed in a large screen of US, Nordic, and German amyotrophic lateral sclerosis/frontotemporal dementia cohorts. Neurobiol Aging.

[11] Chen Y, Zheng ZZ, Huang R, Chen K, Song W, Zhao B, Chen X, Yang Y, Yuan L, Shang HF (1922). PFN1 mutations are rare in Han Chinese populations with amyotrophic lateral sclerosis. Neurobiol Aging.

[12] Smith BN, Vance C, Scotter EL, Troakes C, Wong CH, Topp S, Maekawa S, King A, Mitchell JC, Lund K (2015). Novel mutations support a role for Profilin 1 in the pathogenesis of ALS. Neurobiol Aging.

[13] Fratta P, Charnock J, Collins T, Devoy A, Howard R, Malaspina A, Orrell R, Sidle K, Clarke J, Shoai M (2014). Profilin1 E117G is a moderate risk factor for amyotrophic lateral sclerosis. J Neurol Neurosurg Psychiatry.

[14] Freischmidt A, Muller K, Zondler L, Weydt P, Volk AE, Bozic AL, Walter M, Bonin M, Mayer B, von Arnim CA (2014). Serum microRNAs in patients with genetic amyotrophic lateral sclerosis and pre-manifest mutation carriers. Brain..

[15] Olsen JV, Vermeulen M, Santamaria A, Kumar C, Miller ML, Jensen LJ, Gnad F, Cox J, Jensen TS, Nigg EA (2010). Quantitative phosphoproteomics reveals widespread full phosphorylation site occupancy during mitosis. Sci Signal.

[16] Boopathy S, Silvas TV, Tischbein M, Jansen S, Shandilya SM, Zitzewitz JA, Landers JE, Goode BL, Schiffer CA, Bosco DA (2015). Structural basis for mutation-induced destabilization of profilin 1 in ALS. Proc Natl Acad Sci USA..

[17] Tisdale S, Pellizzoni L (2015). Disease mechanisms and therapeutic approaches in spinal muscular atrophy. J Neurosci.

[18] Giesemann T, Rathke-Hartlieb S, Rothkegel M, Bartsch JW, Buchmeier S, Jockusch BM, Jockusch H (1999). A role for polyproline motifs in the spinal muscular atrophy protein SMN. Profilins bind to and colocalize with smn in nuclear gems. J Biol Chem.

[19] Cauchi RJ (2014). Gem depletion: amyotrophic lateral sclerosis and spinal muscular atrophy crossover. CNS Neurosci Ther.

[20] Kawahara Y, Mieda-Sato A (2012). TDP-43 promotes microRNA biogenesis as a component of the Drosha and Dicer complexes. Proc Natl Acad Sci USA..

[21] Livak KJ, Schmittgen TD (2001). Analysis of relative gene expression data using real-time quantitative PCR and the 2(−Delta Delta C(T)) method. Methods.

[22] Remy I, Michnick SW (2006). A highly sensitive protein-protein interaction assay based on Gaussia luciferase. Nat Methods.

[23] Outeiro TF, Putcha P, Tetzlaff JE, Spoelgen R, Koker M, Carvalho F, Hyman BT, McLean PJ (2008). Formation of toxic oligomeric alpha-synuclein species in living cells. PLoS One.

[24] Jordan M, Wurm F (2004). Transfection of adherent and suspended cells by calcium phosphate. Methods.

